# Clinicopathological features and surgical procedures of adnexal masses with abdominal pain in pediatric and adolescent patients

**DOI:** 10.1186/s13023-024-03101-4

**Published:** 2024-03-21

**Authors:** Qian Liu, Zhiqiang Li, Huimei Zhou, Dongyan Cao, Jiaxin Yang, Keng Shen, Jinghe Lang

**Affiliations:** 1 Department of Obstetrics and Gynecology, Peking Union Medical College Hospital, Peking Union Medical College, Chinese Academy of Medical Sciences, National Clinical Research Center for Obstetric & Gynecologic Diseases, No. 1 ShuaiFu Yuan, Dongcheng District, 100730 Beijing, China; 2 Shandong Cancer Hospital and Institute, Shandong First Medical University, Shandong Academy of Medical Sciences, 250062 Jinan, China

**Keywords:** Abdominal pain, Adolescent, Ovarian mass, Pediatric, Surgery

## Abstract

**Purpose:**

This study investigated the clinicopathological features and surgical procedures of adnexal masses with abdominal pain in pediatric and adolescent patients. Our objective was to better define the clinical presentation of adnexal torsion and to distinguish characteristics of those with torsion and those with an alternate diagnosis.

**Methods:**

Retrospective cohort study of 212 pediatric and adolescent patients was performed who admitted for abdominal pain and presenting with an adnexal mass between March 2012 to December 2019.Medical records were reviewed for age at operation, including presentation of symptoms and signs; the levels of tumor markers; imaging examinations; pathologic findings; the size of masses; treatment; and outcome. Data management and descriptive analyses were performed using SPSS 26.0.

**Results:**

The median age of the patients was 14.5 ± 3.6 years at the operation. 126 (59.4%, 126/212) patients presented with an abrupt onset of abdominal pain. A total of 82.1% (174/212) of the participants underwent adnexal conservative surgery. 179 (84.5%, 179/212) patients underwent laparoscopic surgery with an average tumor size of 7.7 ± 3.4 cm, while 33 patients ( 15.6%, 33/212) underwent laparotomy. Rupture of mass and ectopic pregnancy accounted for 7.5% (16/212) and 0.9%(2/212), respectively. Torsion was responsible for 36.8% (78/212) of all patients. Among the patients with torsion, the symptom of nausea and vomiting was more common among girls without torsion (*P* < 0.0001). 88.5% of the girls with torsion had acute onset of abdominal pain, while 92.3% had persistent pain that could not be relieved or occurred repeatedly, which significantly higher than that in the patients without torsion (*P* < 0.001). 69.2% of patients with torsion had fixed pain sites, compared with 42.2% in patients without torsion (*P* < 0.001). 88.5% of girls with torsion had an ovarian cyst/mass ≥ 5 cm, compared with 75.0% in girls without torsion (*P* = 0.038). 66.7% of girls underwent ovary-preserving surgery, compared with 92.2% in patients without torsion. The most common pathologic types were mature teratoma and simple cyst, accounting for 29.4% and 25.6%, respectively. The multivariate analyses confirmed that mass size greater than 5 cm (OR 4.134, 95% CI: 1.349–12.669,*P* = 0.013), acute onset pain (OR 24.150,95%CI: 8.398–69.444,*P* = 0.000), persistent or recurrent pain (OR 15.911,95%CI: 6.164–41.075,*P* = 0.000) were significantly associated with increased risk of torsion.

**Conclusions:**

Torsion which is a relatively rare event in the pediatric population was not an uncommon condition and responsible for more than one third of all pediatric and adolescent patients presented with adnexal masses and abdominal pain. Pain assessment in children and adolescents is important to distinguish characteristics of those with torsion and those with an alternate diagnosis.Thus, pediatric and adolescent patients particularly with a pelvic mass size greater than 5 cm, acute onset pain, persistent or recurrent pain have a benign cause and not missing the devastating condition that needs emergent attention. Thus, a strategy of earlier and liberal use of Diagnostic Laparoscopy (DL) may improve ovarian salvage.

## Introduction

Ovarian neoplasms (ON) in the pediatric population are rare, with a reported incidence of 2.2 per 100,000 girls per year [1]. The clinical presentation and outcomes of ovarian masses in adolescent and pediatric populations differ from those in adults and require different diagnostic and management approaches [2, 3]. Both benign and malignant neoplasms of the ovary usually need surgical intervention. Surgical treatment in childhood ovarian pathologies is conservative with the objective to preserve development of secondary sex characters and future fertility [4]. For both benign and malignant diseases, the most common symptom is abdominal pain which is a common complaint in girls and young women presenting to a pediatric emergency department (PED). Abdominal pain in previous episodes is a possible indication of torsion and recurrent torsion which differs significantly from appendicitis and other diagnosis.

Our knowledge of ovarian pathologies in children is still far from complete, and much remains to be discovered. Ovarian torsion in the pediatric population is a relatively rare but serious event because of the risk of decreased fertility if it is not treated at an early stage [5]. Torsion was responsible for one third of all operative ovarian cases. In 30% of the patients, there is torsion of a normal adnexa, while the majority of the cases are associated with ovarian pathology [6, 7]. In any case, it is clear that the timely recognition and treatment of adnexal torsion increases the possibility of retaining the ovary and, therefore, can diminish the chance of decreased fertility. Ovarian torsion is defined as twisting of the ovary around an axis consisting of its vascular pedicle, the infundibulopelvic ligament and the tubo-ovarian ligament, and can occur in females of any age. Ovarian torsion remains a challenging diagnosis, often leading to delayed operative intervention and resultant ovarian loss. In earlier studies, a minority of adolescents with suspected torsion actually had a confirmed surgical diagnosis [8], which was similar to that only 46% of women with the preoperative diagnosis of ovarian torsion had the post-operative diagnosis of torsion in adult patients.

The first-line imaging modality in the diagnosis of ovarian torsion is ultrasound [9]. Computed tomography (CT), magnetic resonance imaging(MRI) and contrast-enhanced sonography have been reported to accurately identify adnexal torsion [10–13]. However, clinicians and families prefer to limit invasive procedures and radiation exposure. Symptoms and physical exams still play an important role in the diagnosis of torsion. Previous studies have highlighted the importance of symptoms in diagnosing ovarian torsion, particularly acute, severe abdominal pain, nausea and vomiting [14, 15]. Lawrence et al. emphasized ovarian torsion as clinical diagnosis, and imaging must be interpreted, with caution and consideration of the patients overall presentation and clinical condition [16]. Patients with ovarian tumors often complain of acute or chronic abdominal pain, vomiting, nausea, increased abdominal volume, or other symptoms induced by compression of the surrounding organs [4]. The present study found that either acute or chronic abdominal pain was the most frequent symptom. A lesion, either a mass or a cyst, becomes the most important underlying etiology of torsion. However, the incidence of torsion and the condition of health care in pediatric patients and adolescents with the presence of an adnexal mass and abdominal pain have seldom been reported [17]. This study aimed to investigate the difference in outcomes with diagnostic laparoscopy for all girls presenting with abdominal pain and a pelvic mass.

## Materials and methods

**Fig. 1:**
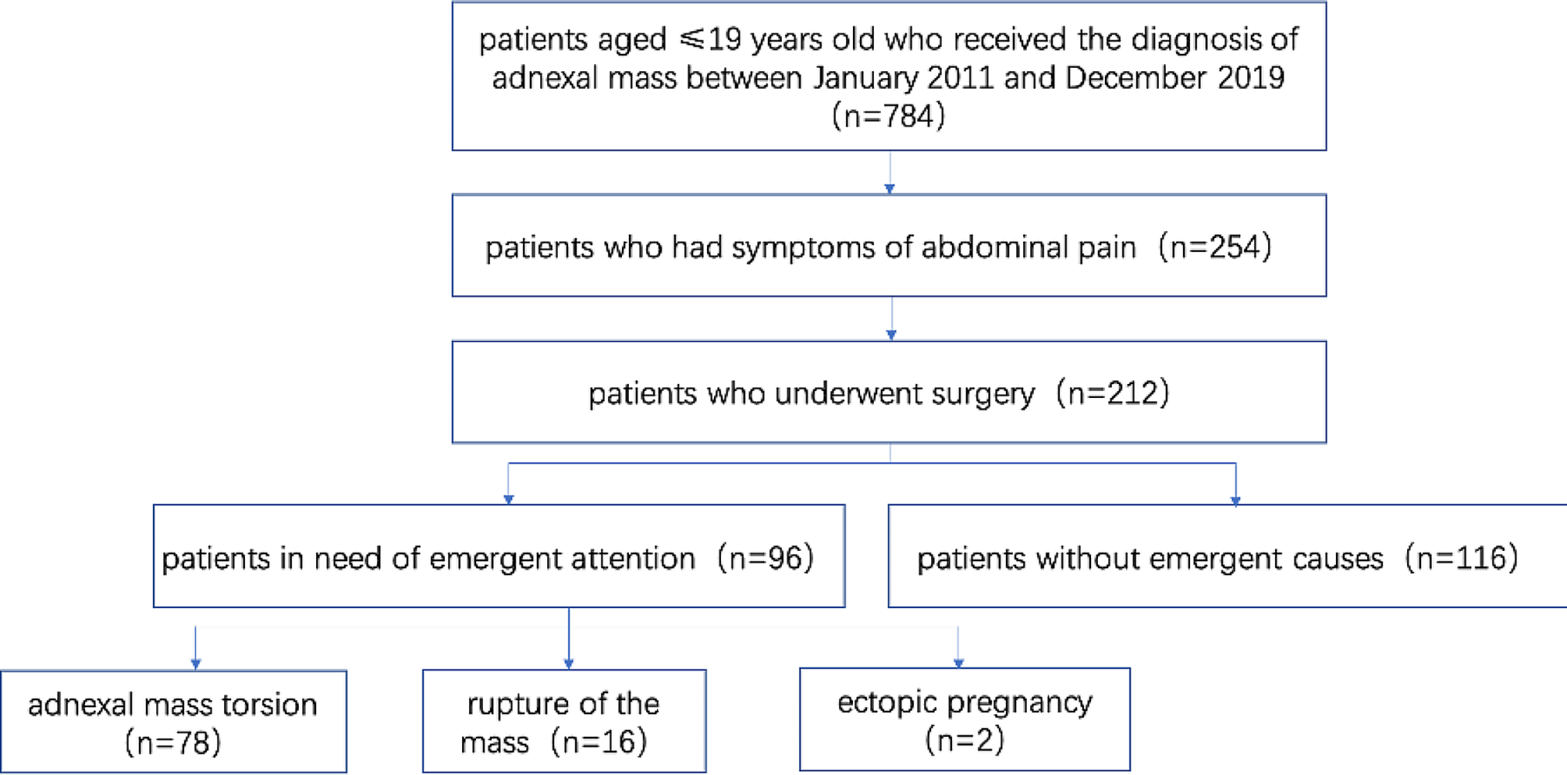
Flowchart of patients included in the study

We conducted a retrospective chart review at Peking Union Medical College Hospital(PUMCH), and 212 patients were queried from March 2012 to December 2019 in the Department of Obstetrics and Gynecology. The study was approved by the Institutional Review Board of Peking Union Medical College Hospital. Informed consent to participate in the study was obtained from all participants. The flowchart of the study is shown in Fig. 1. Medical records were reviewed for age at operation, including presentation of symptoms and signs; the levels of tumor markers (α-fetoprotein (AFP), β-human chorionic gonadotrophin (β-HCG), CA125 and CA199). We reviewed all imaging examinations including the results of ultrasonography and computed tomography (CT) scans. We reviewed all operation records, pathologic findings and the size of masses as documented at surgery or in the pathology record. The qualitative assessment of pain includes pain quality, location and extent, duration, degree, and neurophysiological function was conducted in the study (Table 1).

**Table 1:** Reference ranges of lab tests

Lab tests	Unit	Reference ranges
AFP	ng/ml	≤ 20.0
β-HCG	IU/L	≤ 6.0
CA125	U/ml	≤ 35.0
CA199	U/ml	≤ 34.0

Statistical analyses were performed using Student’s t test as appropriate. A *P* value of 0.05 or less was regarded as statistically significant. All continuous data are expressed as the mean ± SD. Univariate and multivariate logistic regression was used to assess factors associated with torsion. Variables associated with torsion and at an alpha of 0.05 or less were included in a logistic regression model. The odds of torsion for each of the risk factors was computed and reported with the 95% confidence interval (CI) and *P* value.

## Results

### Clinical characteristics of all girls presenting with abdominal pain and a pelvic mass

212 patients were surgically evaluated for ovarian lesions with a symptom of abdominal pain. At the operation, the median age of the patients was 14.5 ± 3.6 years; 155 patients (73.1%, 155/212) had regular menstruation, 20 patients (9.4%, 20/212) had irregular menstruation, and 37 patients (17.5%, 37/212) were premenarchal.

Further, 126 (59.4%, 126/212) patients presented with an abrupt onset of abdominal pain. In addition to pain, the patients presented with other symptoms and objective findings. The incidence rate of vomiting, nausea, fever, diarrhea, frequent urination, and abnormal vaginal bleeding was 26.9%, 6.6%, 3.3%, 0.9%, 1.8%, respectively. One patient presented with the clinical characteristics of precocious puberty.

Pelvic abdominal ultrasound(US) was the priority imaging modality used in all the patients (100%, 212/212) to characterize the ovarian disease as predominantly cystic lesions or lesions with solid components. The US examination showed an adnexal mass in all patients: cystic ovarian masses in 188 (88.7%), lesions with solid components in 24 (11.3%). CT scan or MRI was performed for 39 patients (18.4%) to exclude suspected malignant pathologies. The serum levels of the tumor markers CA-125, CA-199, α-fetoprotein, and β-human chorionic gonadotropin were detected in 105 patients. The levels of tumor markers were abnormally elevated in 26 patients.

### Surgical procedure for patients and pathology review

Among the 212 patients, 179 (84.5%, 179/212) underwent laparoscopic surgery with an average tumor size of 7.7 ± 3.4 cm, with no intraoperative or postoperative complications. The average tumor size of 33 patients (15.6%, 33/212) who underwent laparotomy was 12.9 ± 5.6 cm. The mean tumor size of patients undergoing laparotomy was significantly higher than that of patients undergoing laparoscopic surgery (*P* = 0.000). A total of 82.1% (174/212) of the participants underwent adnexal conservative surgery.

The pathologic examination demonstrated the adnexal mass with abdominal pain being mature cystic teratoma (33.5%, 71/212), simple cyst (18.9%, 40/212), mucinous or serous cystadenoma (11.3%, 24/212), corpus luteum cyst (9.0%, 19/212)( cysts became rupturing and hemorrhagic (bleed),while causing severe pelvic pain), para-ovarian cyst (8.0%, 17/212), endometriosis cyst (8.0%, 17/212), malignant germ cell tumor (3.8%,8/212), and sexual cord stromal cell tumor (3.3%, 7/212). Among complex lesions suspected as malignancy indicated by preoperative ultrasound in 14 patients, 6 were confirmed to be malignant germ cell tumors and 3 were sex cord stromal tumors.

Eight cases of malignant germ cell tumors were found, including four cases of endodermal sinus tumors, one case of mixed germ cell tumors (endodermal sinus tumor complicated with immature teratoma tumor), two cases of immature teratoma, and one case of dysgeminoma. The postoperative treatment of the eight cases was supplemented with three to six cycles of PEB/PEV chemotherapy. Further, seven cases of sex cord stromal cell tumors were found, including four cases of juvenile granulosa cell tumors, one case of malignant steroid cell tumors, and two cases of moderately differentiated Sertoli-Leydig cell tumors. Juvenile granulosa tumors and malignant steroid cell tumors were treated with adjuvant chemotherapy after the surgery.

### Clinical characteristics comparing patients with torsion and those with an alternate diagnosis

Of the 212 patients, 78 (36.9%) cases had adnexal torsion, including adnexal mass, enlarged ovaries, and fallopian tubes; 16 (7.5%) cases had mass rupture; and 2 (0.9%) had ectopic pregnancy; one had pyosalpinx and one had appendicitis (Table 2). The patients were divided into two groups (mass rupture and ectopic pregnancy were excluded from the cohort): the torsion (TO, *n* = 78) group and the non-torsion (non-TO, *n* = 116) group. We compared the clinical presentation between the patients with torsion and those without torsion. The median age for girls with torsion was 14.0 y, compared with 14.9 y for girls with a mass but no torsion (*P* >0.05). The proportion of patients presented with acute onset pain, persistent or recurrent pain, and duration of pain less than 3 months was significantly higher in the TO group than in the non-TO group (*P* < 0.001). 69.2% of patients with torsion had fixed pain sites, compared with 42.2% in patients without torsion (*P* < 0.001). 23.1% of patients with torsion were pre-menarche, compared with 11.2% in girls without torsion (*P* = 0.044). The proportion of patients with age ≤ 11 years in the TO group was significantly higher than that in the non-TO group (*P* = 0.038). The symptom of nausea and vomiting was more common among girls with torsion (*P* < 0.0001). 88.5% of girls with torsion had an ovarian cyst/mass ≥ 5 cm, compared with 75.0% in girls without torsion (*P* = 0.038), but there was no significant difference in the size of the lesion when evaluated continuously (median with torsion 8.8 cm versus median without torsion 8.7 cm; *P* = 0.884) (Table 3).

**Table 2:** Final diagnosis of patient needing emergent attention

Final diagnosis by laparoscopy	Number
Adnexal torsion	78
Adnexal mass	72
Enlarged ovary	3
Fallopian tube	3
Mass rupture	16
Ectopic pregnancy	2
Pyosalpinx	1
Appendicitis	1

**Table 3:** Clinical characteristics in the two groups

	TO (*n* = 78)	non-TO (*n* = 116)	*P* value
**Age (year)**	14.0 ± 3.9	14.9 ± 3.2	0.111
**Menarche**			0.044
Post-menarche	60(76.9%)	103(88.8%)	
Pre-menarche	18(23.1%)	13( (11.2%)	
**Onset of pain**			0.000
Acute onset	69(88.5%)	45(38.8%)	
Non-acute onset	9(11.5%)	71(61.2%)	
**Duration of pain**			0.000
≤3 months	76(97.4%)	87(75.0%)	
>3 months	2(2.6%)	29(25.0%)	
**Outcome of pain**			0.000
Persistent or recurrence	72(92.3%)	47(40.5%)	
Spontaneous remission	6(7.7%)	69(59.5%)	
**Complain of nausea and vomiting**			0.000
No	39(50.0%)	89(76.6%)	
Yes	39(50.0%)	27(23.3%)	
**Fever**			0.413
Yes	2(2.6%)	5(4.3%)	
No	76(97.4%)	111(95.7)	
**Location**			0.000
Right or left quadrant	54(69.2%)	49(42.2%)	
Not accurate site and/or referred pain	24(30.8%)	67(57.8%)	
**Ultrasound**			0.527
Cystic	70(87.5%)	104(89.7%)	
Solid	8(2.1%)	12(1.7%)	
**Surgical approach**			0.000
Cystectomy	52(66.7%)	105(90.5%)	
Oophorectomy and/or salpingectomy	26(33.3%)	11(9.5%)	
**Tumor size**	8.8 ± 3.4	8.7 ± 5.0	0.884
**Tumor size(Median)**			0.038
<5 cm	9(11.5%)	29(25.0%)	
≥5 cm	69(88.5%)	87(75.0%)	
**Laterality**			0.434
Left	33(42.3%)	49(42.2%)	
Right	43(55.1%)	58(50.0%)	
Bilateral	2(2.6%)	9(7.8%)	

Among the 78 patients with adnexal torsion, 72 patients had adnexal mass. The average diameter of the adnexal mass was 8.7 ± 3.1 cm; in 97.2% of patients, it was larger than 5 cm. The average torsion degree was 653.2 ± 419. Torsion occurred on the right side in 45(57.7%)patients and 33 (42.3%) cases on the left. The cysts were bilateral, and torsion occurred on right side in two patients. The most common pathologic types were mature teratoma and simple cyst, accounting for 29.4% and 25.6%, respectively, followed by para-ovarian cyst, serous/mucinous cystadenoma, and endometriosis cyst. One patient presented with torsion of enlarged ovarian, three patients with fallopian tube torsion, three cases with malignant germ cell tumor, and one patient with sex cord stromal tumor. 66.7% of girls underwent ovary-preserving surgery, compared with 92.2% in patients without torsion. Further, 23 patients (29.5%) underwent adnexectomy due to ovarian necrosis. Patients with necrosis had more torsion cycles than those without necrosis, the difference was not statistically significant (*P* = 0.021). The proportion of patients with necrosis whose pain duration was more than 72 h was higher than that of patients without necrosis (*P* = 0.700). Three patients underwent salpingectomy for tubal torsion in the girls with torsion (Table 4).

**Table 4:** Surgical method for patients with adnexal torsion

	TO (*n* = 78)	non-TO (*n* = 116)
**Adnexectomy**	24	6
Mature cystic teratoma	6	1
Serous/mucinous cystadenoma	1	1
Simple cyst	9	2
Para-ovarian cyst	5	0
Corpus luteum cyst	0	0
Enlarged ovary	2	0
Sexual cord stromal cell tumor	1	2
**Cystectomy**	**49**	**107**
Mature cystic teratoma	17	47
Serous/mucinous cystadenoma	3	19
Simple cyst	11	18
Para-ovarian cyst	7	5
Corpus luteum cyst	5	5
Endometriosis cyst	3	11
Enlarged ovary	1	1
Malignant germ cell tumor	2	1
**Salpingectomy**	**3**	**0**
Tubal torsion	3	0
**Adnexectomy ± greater omentum resection + biopsy**	**2**	**3**
Granulosa cell tumor	1	0
Malignant germ cell tumor	0	2
Immature teratoma	1	1

### Factors associated with adnexal mass torsion

Univariate and multivariate logistic regression was used to assess factors associated with adnexal mass torsion. The multivariate analyses confirmed that mass size greater than 5 cm, acute onset pain, persistent or recurrent pain were significantly associated with increased risk of torsion (Table 5). Notably, patients with mass size greater than 5 cm had 4.1 times the odds of developing torsion in the presence of a mass as compared with those with smaller masses (95% CI: 1.349–12.669). Patients presented with persistent or recurrent pain was associated with 24.2 times the odds of torsion (95%CI: 8.398–69.444), and acute onset pain was associated with 15.9 times the odds of torsion (95%CI: 6.164–41.075).

**Table 5:** univariate and multivariate analysis of risk factor for torsion

	Univariate analysis			Multivariate analysis		
Risk factor	Odds ratio	95% CI	*P*-value	Odds ratio	95% CI	*P*-value
Size ≥ 5 cm	2.556	1.1355.755	0.024	4.134	1.349–12.669	0.013
Acute onset pain	12.096	5.497–26.617	0.000	15.911	6.164–41.075	0.000
Fixed pain sites	3.077	1.679–5.638	0.000	-	-	-
Persistent or recurrent pain	17.617	7.080-43.836	0.000	24.150	8.398–69.444	0.000
Nausea and vomiting	3.296	1.776–6.118	0.000	-	-	-

## Discussion

Acute abdominal pain in children and adolescents poses a diagnostic challenge due to various underlying causes. The age of the patient can help focus the differential diagnosis. Since the differential for acute abdominal pain is wide and may vary by clinical specialty, it is critical to consider a multi-disciplinary approach when girls present with non-specific symptoms such as pain, nausea, and vomiting. Accompanying symptoms and signs are inconsistently present, especially in toddlers and school-aged children; however, these signs/symptoms can guide the selection of appropriate diagnostic tests, imaging, and definitive treatment. Thus, accurate and timely diagnosis is sought to avoid both inappropriate treatment (as many causes of acute abdominal pain need a non-surgical treatment) [18, 19] and diagnostic delays (which increase morbidity). This article reviews common benign causes of abdominal pain as well as some of the cannot-miss emergent causes. We presented a large cohort of girls diagnosed with adnexal masses with abdominal pain and analyzed their clinicopathological characteristics and the difference in outcomes. We further analyzed the risk factors such as pelvic mass size greater than 5 cm, acute onset pain, persistent or recurrent pain for adnexal mass torsion to determine which child likely has a benign cause and not missing the devastating condition that needs emergent attention.

### Different outcome of girls presenting with acute abdominal pain and a pelvic mass

Adnexal mass torsion and rupture are typically not primary considerations for differential diagnoses of a child with acute abdominal pain, given previous estimates that torsion accounts for only 2.7% of acute abdominal pain in the pediatric population [20]. However, ovarian disorders such as cyst rupture and torsion must be considered when evaluating girls with acute-onset abdominal pain [14, 21]. It is difficult to determine the exact risk of acute complications in patients with an asymptomatic ovarian mass because the torsion and rupture of the mass are usually diagnosed when a symptomatic patient undergoes surgery. Oltmann [17] retrospectively reviewed 328 patients with ovarian operative cases at a free-standing children’s hospital over 15 years, which indicated that torsion was responsible for one third of all cases. However, they both failed to further investigate the difference in outcomes presenting with acute abdominal pain and a pelvic mass. In our study, almost half of the patients had acute complications of the adnexal mass confirmed by surgery. Rupture of mass and ectopic pregnancy accounted for 7.5% and 0.9%, respectively. Torsion was responsible for 36.8% of all patients, which was greatly higher than the previous study. Therefore, torsion is not an uncommon condition in patients with adnexal masses and abdominal pain, which should be considered in all girls presenting with abdominal pain.

### Pain assessment in children and adolescents is important to distinguish characteristics of those with torsion and those with an alternate diagnosis

Ovarian torsion remains a diagnosis of exclusion despite technologic advances in imaging. Symptoms of lower abdominal pain, nausea, and vomiting, commonly seen in patients with acute ovarian torsion, overlap with symptoms from numerous other etiologies. Intensity, nature, location, and duration of pain can vary greatly from patient to patient with ovarian torsion [17]. Abdominal pain may be intermittent and recurrent, accompanied by vomiting [14, 22].Our study examined the group of girls who presented with pain, but did not have the diagnosis of torsion and summarized the pain characteristics. We distinguish characteristics of those with torsion and those with an alternate diagnosis. The study showed that pain assessment is particularly important, especially in patients who presented with acute pain characterized by emergency pain onset, persistent pain that could not be relieved or recurrent pain. Among the patients with torsion, the symptom of nausea and vomiting was more common among girls without torsion (*P* < 0.0001). 88.5% of the girls with torsion had acute onset of abdominal pain, while 92.3% had persistent pain that could not be relieved or occurred repeatedly, which significantly higher than that in the patients without torsion. 69.2% of patients with torsion had fixed pain sites, compared with 42.2% in patients without torsion (*P* < 0.001). Multivariate analysis confirmed that patients presented with persistent or recurrent pain was associated with 24.2 times the odds of torsion (95%CI: 8.398–69.444), and acute onset pain was associated with 15.9 times the odds of torsion. When the girls presented with adnexal mass is combined with the aforementioned pain characteristics, clinicians should be on high alert for the possibility of torsion and decide the need for emergency diagnostic laparoscopy according to the patient’s general status. However, in this study, we found that many patients did not seek medical attention as quickly as possible when they had pain symptoms. Also, the average time from the onset of pain symptoms to the consultation was 91 h, suggesting the importance of early consultation to exclude adnexal pathological lesions requiring emergent attention. Pain assessment in children and adolescents is difficult, especially in infants and children. The study failed to include the degree of pain in children. Hence, qualitative assessment of pain includes pain quality, location and extent, time, degree, and neurophysiological function should be included in future studies to accurately assess pain characteristics and provide a basis for guiding clinical decision-making.

### Risk factors for adnexal torsion in girls presenting with abdominal pain and a pelvic mass

Adnexal torsion is usually unilateral and presents as simple ovary or fallopian tube torsion, but simultaneous ovary and fallopian tube torsion is most common. Further, 46% of adolescent cases have only ovarian torsion without ovarian cysts [23]. In this study, the incidence of ovarian torsion without ovarian cysts was 3.8%, which was lower than the data reported in the literature. The size and type of mass seem to be important contributing factors. Three retrospective reviews found that at least 80% of ovarian torsion occurred when the ovary was enlarged by > 5 cm [24–26]. The most common histologic subtypes were benign hemorrhagic cysts, mature teratomas, and serous cystadenomas [24]. Ovarian malignancy is rarely associated with ovarian torsion (< 2% of cases). Our results were consistent with the data reported in the literature, indicating that the size and type of the mass were the influencing factors for adnexal mass torsion. In our study, the average tumor size of torsion was 8.8 cm, and patients with mass size greater than 5 cm had 4.1 times the odds of developing torsion in the presence of a mass as compared with those with smaller masses. The probability of ovarian malignant tumor torsion was 6.4%, and the most common pathologic types were mature teratoma and simple cyst, accounting for 29.4% and 25.6%, respectively. The reason for torsion might be related to the weight and center of gravity of the mass and the relatively long ovary tube and ligaments during the development of children and adolescence. In this study, 23 patients (29.5%) underwent adnexectomy due to ovarian necrosis. Patients with necrosis had more torsion cycles than those without necrosis, and the proportion of patients with pain lasting more than 72 h was higher than that of patients without necrosis. The occurrence of necrosis might be related to the number of turns, tightness, and duration of torsion.

### The timing and method of the surgery are crucial factors that need careful consideration

The surgical indications for adnexal mass in pediatric and adolescent patients, though not absolute, include cysts greater than 5 cm in diameter, a failure of the cyst to resolve or decrease in size spontaneously, complex or solid cysts indicative of suspected malignancy, severe persistent abdominal pain, and complications such as ovarian torsion, hemorrhage, or infarction [27, 28]. In our study, we enrolled patients diagnosed with an adnexal mass accompanied by abdominal pain, and the average size of the adnexal mass was 8.6 cm at the time of admission. Among the 212 patients, benign and malignant tumors accounted for 92.9% and 7.1%, respectively. The most common pathological type was mature teratoma cyst, accounting for 33.5%. Although benign masses are more common in patients with adnexal masses accompanied by abdominal pain, the occurrence of acute complications such as torsion can greatly increase the risk of ovarian resection. Thus, pediatric and adolescent patients particularly with a pelvic mass size greater than 5 cm, acute onset pain, persistent or recurrent pain have a benign cause and not missing the devastating condition that needs emergent attention and diagnostic laparoscopy. It is important to perform a careful and thorough evaluation before proceeding with any surgical treatments in patients with suspected malignant lesion.

## Conclusions

Our research included an evaluation of girls with similar presentation, but without torsion, to improve accuracy of the diagnosis of adnexal torsion. We showed that pain assessment was important, especially in girls particularly with a pelvic mass size greater than 5 cm. If the abdominal pain presented with acute pain which characterized by emergency pain onset, and persistent pain that could not be relieved or recurrent, the surgical emergency should be underwent. Clinicians should remain vigilant of the possibility of the adnexal mass combined with acute complications and decide the need for emergency diagnostic laparoscopy according to the patient’s general status. We hope that our results facilitate rapid surgical management to those for whom the suspicion of torsion is high. For patients with suspected malignant lesions, the lesions should be comprehensively evaluated preoperatively.

## Data Availability

Patient-level data can be made available from the corresponding author after discussion with the trial management committee.

## Abbreviations

CI: confidence interval
CT: Computed tomography
DL: Diagnostic laparoscopy
MRI: Magnetic resonance imaging
non: TO non-torsion
ON: Ovarian neoplasms
PEB: Cisplatin Etoposide Boromycin
PEV: Cisplatin Etoposide Vincristine
PED: pediatric emergency department
PUMCH: Peking Union Medical College Hospital
TO: torsion
US: ultrasound
